# Bridging the gap: Work as a treatment goal in healthcare. An innovative approach at Radboudumc

**DOI:** 10.1177/10519815251319239

**Published:** 2025-02-20

**Authors:** Desiree JS Dona, Theo F Senden, Marlies EWJ Peters, Chelsea Walden

**Affiliations:** 1 Department of Primary and Community Care, Radboud University Medical Center, Nijmegen, The Netherlands; 2 Department of Primary and Community Care, Radboud University Medical Center, Nijmegen, The Netherlands; 3 Department of Human Resources/Occupational Health Services (retired), Radboud University Medical Center, Nijmegen, The Netherlands

**Keywords:** chronic disease, work-oriented care, patient-oriented care, integrated care, occupational health physician, multidisciplinary approach, healthcare collaboration, workplace reintegration

## Abstract

**Background:**

The number of people with chronic conditions among (potential) workers is expected to increase significantly in the coming years. These individuals experience problems with study, work and income. Both study and work are important to patients and contribute significantly to quality of life.

**Objective:**

For this reason, attention to study and work has been integrated into regular care across a number of patient groups at Radboud University Medical Center (Radboudumc).

**Methods:**

Here, work is considered a treatment goal as part of the individuals care plan from diagnosis onwards. Work-oriented care takes shape through the training of nurse specialists, deployment of clinical occupational physicians and guidance to patients.

**Results:**

An initial evaluation showed that patients and healthcare professionals believed that work-oriented care added value for both parties.

**Conclusıons:**

As such, it is important for the future that work-oriented care is structurally embedded in the organization and financing of regular care.

## Introduction

In the Netherlands, one-third of the population has a chronic health condition, a figure set to rise to 40% by 2030.^1^ Conditions are considered chronic when they are long-lasting, impact daily functioning and require medical intervention. Examples of common chronic conditions in the Netherlands include cancer, cardiovascular disease, gastrointestinal diseases (e.g., inflammatory bowel disease), neurological disorders (e.g., Parkinson's disease and cerebral infarct), diabetes and post-acute infection syndromes such as long-covid and post-Lyme. Most chronic conditions develop during working age (25–67 years), with 38% of Dutch workers diagnosed with one or more chronic condition.^2^ While the Netherlands has high employment rates, it finds itself in the middle bracket when it comes to supporting workers with chronic disease.^3^ For these individuals, retaining or returning to work is problematic, while job retention being important for quality of life, structure, meaning, income and financial security.^4–8^ Ramifications extend beyond individual well-being, including societal loss of working force, talent and expertise.^3,6,9^ Regular care is rarely work-oriented. Focus is placed solely on treatment of the medical condition and not on the (prevention of) impact on work(ability) and participation. Occupational health care is only accessible to workers in permanent/temporary employment contracts (approximately 50%).^10^ Further, there is little cooperation between occupational physicians, general practitioners and medical specialists (MS), despite several improvement initiatives in recent decades.^10^ Barriers to effective cooperation include organisation of care (separation between health care and occupational health care), legislation (occupational physicians can only access medical information with written permission of the patient) and differences in financing streams between medical care (financed by the Health Insurance Act) and professionals within the domain of work (paid privately by employers/self-employed individuals). As such, there is growing imperative to develop innovative solutions to bridge the gap between healthcare and work^10,11^ in order to provide holistic support aimed at integration of work participation as a treatment goal for those living with a chronic condition.

## The Work-Oriented Care Initiative at Radboudumc

To improve focus on work participation in regular care, Radboud university medical centre (Radboudumc) began systematically developing and implementing work-oriented care across a number of departments a decade ago.^12–14^ Here, retainment or restoration of social functioning and work participation is a treatment goal and is also used as a recovery-promoting intervention.

Clinical occupational physicians (COPs) are experienced occupational physicians with disease-specific expertise that are embedded in the multi-disciplinary health care team within Radboudumc. The COPs act as a crucial source of occupational medicine expertise in the care team and a link between healthcare and work domains, facilitating integration of work ability and work participation as a treatment goal from diagnosis. Nurse specialists (NS) within treatment teams also have an important role, focusing on the psychosocial and societal implications of illness and treatment.^12,14^ NS's have been trained and coached to identify work related issues and provide basic work-oriented care, refer to the COP when required and monitor the work-oriented intervention plan alongside the patient.

The aim is to support patients through all stages of their patient journey, from diagnosis to the workplace. This allows patients to maintain work and income, in a way that suits their needs and capabilities sustainably. More than 2000 patients with chronic conditions (stroke, Parkinson's, IBD, Lyme, Onco- and Hematology) have now been supported at Radboudumc.

## Work-Oriented Care Method: the role of the COP

At Radboudumc, the COP is embedded within the treatment team across a number of departments.^15^ If highlighted as requiring support, patients are referred for consultation with COPs by the MS/NS during multi-disciplinary team consultations.^15^ During the consultation, the COP carries out a multifactorial problem analysis (based on the International Classification of Functioning, Disability and Health model,^16^ assessing work ability and considering factors in all ICF-domains that may facilitate or impede working ability and participation.^15^ The ICF model allows the COP to understand the patients participation problem through the biopsychosocial approach; considering both medical, environmental and psychosocial factors.^15,16^ Together with the patient the COP creates an intervention plan addressing the identified facilitating and impeding factors for work retainment, tailored to the patient's wishes and achievable goals, including advice for healthcare, work and/or social domains.^15^ The COP coordinates with and/or refers to relevant professionals in both health care and work domains when required. In this way, medical information and information about the workplace can be shared and coordinated.^15^ This may involve connecting with the patients occupational physician (if patient is employed) or a job coach to deploy work interventions that help the patient reach their personal goals (e.g., adjustment or change of work).^15^ The COP is also able to provide advice about re-integration approach, regulations, finances and benefit systems.^15^ Throughout the process, the NS monitors the patient and if necessary, consults or refers back to the COP.^15^ The COP adds disease-specific occupational medical expertise to the process and is the linking-pin between the health care and work domain.^15^ The organisations occupational physician has and keeps a formal responsibility and authority to judge the employability and advice the employer (e.g., about adjustment of work conditions and working hours).

## Evaluation and impact

A recent evaluation of the paradigm shifting work-oriented care model at Radboudumc has revealed promising outcomes. Patient surveys demonstrated a substantial impact on experienced work and quality of life, with 90% (n = 129) reporting added value from their interaction with a COP.^12^ These surveys were completed across four patient groups: Stroke, Parkinson's, Onco-Hematology and IBD, with 95% of these patients indicating that the COP had appropriate knowledge of the disease and treatment.

Healthcare professionals also acknowledged the effectiveness of this novel approach, emphasising its alignment with their own responsibilities and the seamless collaboration with the COPs. Interviews with healthcare professionals from Stroke, Parkinson's and Onco-Hematology revealed that while they felt competent to raise the subject of work or study, specific matters such as assessment of work capacity, referrals for work-oriented interventions, regulations and finance really belong to the COP. The NS is seen to have an important role in counselling patients and are able to provide basic work-oriented care after attending training and coaching in a multidisciplinary case consultation, which prevents referral of every patient with questions or problems about employment to the COP.^14^

## Three testimonials from patients

“The COP has initiated a process through which I have followed a rehabilitation process and I have a new job that suits me better in terms of load and load capacity. I am very grateful for the referral to the COP. It was the starting point for a better life, and learning to live better with my disability.”

“I am very happy that I came into contact with the COP through the nursing specialist and the COP helped me by encouraging me and giving me the opportunity to get support with return to work. Now I feel in the middle of life again!”

“In my experience, work-related issues are largely related to the invisible aspects of Parkinson disease. It is precisely these aspects that are confused with character traits in a working environment and are therefore quickly labeled as a functioning problem. Explanation of the COP in the conversation with my manager made the difference between an exit process and the possibility to adjust my work so that I was able to continue working for another 4 years.”

## Three testimonials from medical specialists

“Because the COP has a lot of experience in oncology and understands what an oncological treatment entails, the COP can give applied advice. The own occupational physician often gives a general advice that is insufficiently person-oriented and tailor-made. Due to given handles, the treatment process can also be completed more easily; it is stressful when people have problems with work. Often a patient feels reassured, after only 1 consultation.”

“Once things get better at work, you will see the patient flourish, in their social life and psychosocial well-being and sometimes in their somatic complaints. So that's just fine.”

“I observe that patients have a greater grasp on their situation, besides the work outcome, but they are more in control, comprehend it better, and feel more peace and direction.”

## Developments and advancements

A political lobby in 2020, together with the Dutch Federation of Cancer Patient Organisations (NFK), led to a wall-to-wall motion.^17,18^ It called on the Minister of Social Affairs and Employment (SZW) and the Minister of Health, Welfare and Sports (VWS), to arrange appropriate, structural financing for COP care for workers with cancer.

In 2021, the minister replied to the House of Representatives about next steps; further developing and professionalising of the COP profile and practice, evaluation of the fit of COP-care to the requirements for regular financing.^19^ This was the starting point for discussions in a joint project group of occupational health professionals and departmental officials to elaborate and adjust this statement to reach a relevant and realistic formulation of a project assignment. As such, the project ‘Exploration and development of a clinical occupational care model for cancer patients, including a scenario for financing’ was commissioned by SZW in collaboration with VWS that started in December 2023. The developed care model should also be applicable as a blueprint for other patient groups.

To promote work participation and job retention as a treatment goal in health care, the Medical Specialists Knowledge Institute (KIMS/ MSKI) has developed a Generic Module Labor Participation for medical specialist guidelines on behalf of the Federation of Medical Specialists (FMS), co-initiated by the Dutch Patient Federation. The guideline module was approved and published in May 2024.^20^ In the coming years the implementation will be supported by training of healthcare professionals, development of implementation aids, dissemination of good examples. Facilitating access to the expertise of a COP is an important part of the implementation process.

The Generic Module Labor Participation is in line with principles of appropriate care, aimed, among other things, at limiting healthcare costs, more attention to promoting health and preventing the consequences of illness. The aim is to provide MS with concrete tools to promote job retention or return to work from diagnosis onwards to limit unnecessary loss of work and income. The guideline module provides also a preview of a future comprehensive work-oriented care model that will be the basis of the before mentioned project.

Since 2019, nursing students and medical students have been receiving education about work as a treatment goal. An e-module has been created together with the Radboud Health Academy. Occupational health expertise training for nurses and physician assistants has also been set up. Outside Radboudumc, knowledge and experience are shared at conferences, in national forums, in working groups and among patient associations.

## Future vision

The vision for work-oriented care aligns with the pursuit of holistic person-oriented healthcare. The use of technology with regard to communication, cooperation and workflow support enables Radboudumc COPs to organise the care surrounding the patient in regional cross-domain care networks, together with partners in the healthcare, social and work/reintegration domains. This integrated and lineless approach is in line with government policy on regional health care organization, connecting health care and social domains, as laid down in the nationwide Integral Care Agreement (IZA).^21^ In conclusion, the integration of work-oriented care is targeting the improvement of societal participation and the patient's quality of life and represents a pivotal step towards a more inclusive healthcare approach in the Netherlands, addressing the unique challenges faced by those with chronic health conditions.
